# Resectable pancreatic adenocarcinoma neo-adjuvant FOLF(IRIN)OX-based chemotherapy - a multicenter, non-comparative, randomized, phase II trial (PANACHE01-PRODIGE48 study)

**DOI:** 10.1186/s12885-018-4663-4

**Published:** 2018-07-24

**Authors:** Lilian Schwarz, Dewi Vernerey, Jean-Baptiste Bachet, Jean-Jacques Tuech, Fabienne Portales, Pierre Michel, Antonio Sa Cunha

**Affiliations:** 1 Department of Digestive Surgery, Hôpital Charles Nicolle, Rouen, France; 2 UNIROUEN, UMR 1245 INSERM, Rouen University Hospital, Department of Genomic and Personalized Medicine in Cancer and Neurological Disorders, Normandie Univ, F-76000 Rouen, France; 3 Methodological and Quality of Life in Oncology Unit, INSERM UMR 1098, University Hospital of Besançon, Besançon, France; 4 Department of Hepato-Gastroenterology, Hôpital Pitié Salpêtrière, Paris, France; 5 Department of Digestive Oncology, Institut régional du Cancer de Montpellier (ICM) - Val d’Aurelle, Montpellier, France; 6 Department of Hepato-Gastroenterology, Rouen University Hospital, Rouen, France; 7 Department of Hepatobiliary and Pancreatic Surgery and Liver Transplantation, Paul Brousse Hospital, Villejuif, France

**Keywords:** Resectable pancreatic adenocarcinoma, Neoadjuvant chemotherapy, Surgery, mFOLFIRINOX, Randomized study

## Abstract

**Background:**

At time of diagnosis, less than 10% of patients with pancreatic adenocarcinomas (PDAC) are considered to be immediately operable (i.e. resectable). Considering their poor overall survival (OS), only tumours without vascular invasion (NCCN 2017) should be considered for resection, i.e. those for which resection with disease-free margins (R0) is theoretically possible in absence of presurgery treatment. With regard to high R1 rates and undetectable locoregional and/or metastatic spreading prior to surgery explain (at least in part) the observed 1-year relapse and mortality rates of 50 and 25%, respectively. Today, upfront surgery followed by adjuvant chemotherapy is the reference treatment in Europe. The main limitation of the adjuvant approach is the low rate of completion of the full therapeutic sequence. Indeed, only 47 to 60% patients received any adjuvant therapy after resection compared to more than 75% for neoadjuvant therapy. No previous prospective study has compared this approach to a neoadjuvant FOLFIRINOX or FOLFOX chemotherapy for resectable PDAC.

**Methods:**

PANACHE01-PRODIGE48 is a prospective multicentre controlled randomized non comparative Phase II trial, evaluating the safety and efficacy of two regimens of neo-adjuvant chemotherapy (4 cycles of mFOLFIRINOX or FOLFOX) relative to the current reference treatment (surgery and then adjuvant chemotherapy) in patients with resectable PDAC. The main co-primary endpoints are OS rate at 12 months and the rate of patients undergoing the full therapeutic sequence.

**Discussion:**

The “ideal” cancer treatment for resectable PDAC would have the following characteristics: administration to the highest possible proportion of patients, ability to identify fast-progressing patients (i.e. poor candidates for surgery), a low rate of R1 resections (through optimisation of local disease control), and an acceptable toxicity profile. The neoadjuvant approach may meet all these criteria. With respect to published data on the efficacy of FOLFOX and mFOLFIRINOX, these two regimens are potential candidates for neoadjuvant use in the aim to optimising oncological outcomes in resectable PDAC.

**Trial registration:**

ClinicalTrials.gov, NCT02959879. Trial registration date: November 9, 2016.

## Background

Pancreatic duct adenocarcinoma (PDAC) is one of the most lethal forms of cancer, with 5-year overall survival (OS) rates of less than 5% for all stages [1]. It is the fourth-ranked cause of death by cancer and its incidence is steadily increasing in most western countries [2]. At diagnosis, 80 to 85% of patients present locally advanced or metastatic disease, only 5 to 22% of whom can be curatively treated by resection which explains the poor prognosis for PDAC in general [3, 4].

It is established that surgery is the only potentially curative treatment. However, even in resectable PDAC, the oncological results regarding OS and/or health-related quality of life (HRQoL)) need to be optimized. Indeed, in the absence of any adjuvant or neoadjuvant therapy, the long-term outcomes of surgically treated patients are still poor, with a 5-year OS rate of about 10% [5–7].

These very poor oncological results may be due to the presence of micrometastases or minimal residual disease not detectable at the time of surgery or spreading of cancer cells into the portal vein, lymphatic vessels and the peritoneal cavity following surgical manipulation of the tumour. Lymph node involvement (considered as a marker of early tumour dissemination) is found in more than 65% of cases [8]. According to the last American National Comprehensive Cancer Network classification (NCCN 2017) [9], a “immediately resectable” PDAC is defined by no tumor contact with the superior mesenteric vein (SMV) or portal vein (PV) or ≤ 180° contact without vein contour irregularity and no arterial contact.

However, a recent prospective analysis of PDAC resection specimens (using a standardized pathological reporting protocol) has demonstrated that even according to the “redefinition” of margin status, the frequency of positive resection margins (R1) is high (between 61 and 85%) [10–12]. R1 resection is a validated and robust prognostic factor after curative intent resection of PDAC [5–7, 13, 14].

Moreover, pancreatic surgery is associated with a high frequency of post-operative complications [15, 16], and chronic digestive and metabolic sequelae [17]. This is the main argument for optimizing patient selection and using the optimized surgical modality in good candidates in order to prevent palliative resection and to maintain HRQoL).

### Adjuvant chemotherapy: Current standards of care

Randomized trials have suggested that both adjuvant 5-fluorouracil (5-FU) and gemcitabine improve median OS of 2.6–4.5 months and 2-year OS rate of 6 to 10%, when compared with pancreatic resection alone. No significant difference was demonstrated regarding either HRQoL and OS between these two drugs [5–7, 18, 19]. Gemcitabine is today’s drug of choice for adjuvant chemotherapy because better tolerated [7].

Recently, ESPAC 4 trial demonstrated that the combination of gemcitabine and capecitabine allowed a median overall survival of 28 months compared with 25.5 months following gemcitabine alone (*p* = 0.032) [14]. However, disease free survivals (DFS) were not statistically different between the two arms.

Long-term results are still poor, with 5-year OS rates of about 10 and 29% in the presence and absence of adjuvant chemotherapy, respectively [5, 6, 14, 19].

One limitation of the adjuvant approach was the low rate of completion of the full therapeutic sequence due to postoperative complications and poor performance status. Indeed, only 47 to 60% of patients received any adjuvant therapy after resection [20–22]. From an oncological point of view, completion of the full therapeutic sequence (surgery + adjuvant therapy) is well known to be strongly associated with favourable OS among patients with resectable PDAC [22, 23].

In the subset of patients receiving adjuvant treatment, pancreatic cancer exhibits a prominent tendency to recur locally and to metastasize after a short period of time; 1-year OS and DFS rates are 75 and 50%, respectively [5, 6, 24]. The pattern of disease failure corresponds to extrapancreatic dissemination in 63 to 83% of patients and isolated local failure for 17 to 37% of patients [5, 7, 18].

### Oncological treatement accessibility

Despite the theoretical benefits discussed above, there is currently no unambiguous evidence in favour of routine clinical use of neoadjuvant therapy in resectable PDAC. In fact, several single-arm studies have reported heterogeneous results, with median OS and 2-year OS rates ranging from 15 to 35 months and 27 to 55%, respectively [25–31]. These results cannot be compared directly, due to differences in trial design. Nevertheless, the published long-term results are better than those achieved in modern series of patients undergoing surgery alone [5, 24] (median OS: 18–20 months; 2-year OS rate: 40–42%) and appear to be of the same order of magnitude or moderately better than those observed in series of patients treated with adjuvant single-agent chemotherapy [5, 7, 18, 24] (median survival: 21–25 months, 2-year OS rate: 41–48%) or combination chemotherapy [32–34] (median survival: 25.4–32.1 months; 2-year OS rate: 59–80%). A systematic review and meta-analysis of 111 trials confirmed that neoadjuvant treatment does not seem to provide any benefit over adjuvant therapy in resectable PDAC [20].

However, inter-trial comparisons (which already have many serious limitations) are further hampered in this particular case by the fact that the typical population enrolled in adjuvant trials is better selected than those in neoadjuvant trials. For example, 15% of the tumours staged as resectable cannot be resected due to undetected metastatic disease or underestimated vascular involvement, furthermore up to 30% of the patients are not suitable to receive adjuvant therapy because of poor postoperative performance status. Both groups of patients cannot be enrolled into adjuvant trials, thus improving OS by simple patient selection.

An additional argument to support neoadjuvant treatment has been recently published in a report of the extracted data of the American National Cancer Database [35]. Indeed, Mokdad et al. demonstrated that neoadjuvant treatment followed by resection was associated with a significant OS benefit compared to upfront resection in early-stage resected PDAC, with a median survival of 26 months and 21 months, respectively (*p* = 0.01).

### FOLFIRINOX as a potential candidate to improve oncological results

Since the first usage of FOLFIRINOX in the setting of advanced PDAC by Conroy et al. in 2005, and the result of the randomized control trial of the PRODIGE intergroup in metastatic PDAC, this polychemotherapeutic regimen has been used widely [36–39]. Following FOLFIRINOX based treatment, the overall R0 resection rates were about 70–77% [37] and 84–92% [38, 39], respectively for locally advanced and borderline PDAC. In addition, pathologic complete response could be achieved in 7 to 13% in cases.

### FOLFOX as a potential alternative to FOLFIRINOX

Recently, the NCCN stated that Fluoropyrimidines plus oxaliplatin combination therapy can be an acceptable option for patients with gemcitabine refractory PDAC [9].

In a prospective phase II trial that sought to evaluate sequential FOLFOX-6 and gemcitabine followed by appropriate maintenance treatment for advanced pancreatic cancer, the clinical response rate was 44% with an acceptable and manageable toxicity profile [40]. Morever, in patients with gemcitabine refractory PDAC, the combination of oxaliplatin and 5-FU was superior to 5-FU alone or best supportive care in the CONKO-003 trial [41].

Progress is needed, novel drugs have to be tested and alternative strategies evaluated - mainly because adjuvant therapy may not provide any benefit to between a third and a half of patients regarding surgical complications and does not have any impact on the resection margin - one of the main prognostic factors for OS.

## Methods/design

### Study design

**PANACHE-PRODIGE-48** is a prospective open, non-comparative, randomized, multicentre phase II study designed to assess the safety and efficacy of two modes regimens of neoadjuvant chemotherapy (mFOLFIRINOX & FOLFOX) relative to the current reference treatment (surgery follow by adjuvant chemotherapy) for resectable PDAC.

Patients with resectable PDAC (definition based on the NCCN’s) [9] will be randomised to either pancreatectomy and adjuvant chemotherapy or 4 cycles of neoadjuvant chemotherapy with either FOLFOX or mFOLFIRINOX. The patients in the neoadjuvant chemotherapy arms will receive postoperative chemotherapy for 4 months (allowing a 6 months total duration of chemotherapy).

For each patient, treatment time is approximately 7–8 months, with an anticipated maximum period of 9 months.

The primary analysis will be performed on a co-primary endpoint overall survival rate at 12 months after randomisation and the rate of patients undergoing the full therapeutic sequence. An intermediate analysis will be performed once 27 patients evaluable for the co primary endpoint with 12 months of follow-up have been included in each of the 2 arms. The study is planned for a total duration of 5 years. The secondary analyses will be performed 3 years after the last inclusion.

The study is registred on clinicaltrial.gov website (NCT02959879).

### Primary objective

The primary objective of the trial is to evaluate the feasibility and efficacy of two regimens of neoadjuvant chemotherapy. The co-primary endpoints are the observed OS rate at 12 months and the rate of patients undergoing the full therapeutic sequence.

OS is defined as time from randomisation to death from any cause. Alive patients will be censored at the last follow-up [42]

Feasibility is defined as the rate of patients having undergone the full therapeutic sequence (regardless of the study arm). Specific definitions are required in each study arm and so “full therapeutic sequence” are defined as:

- “two or more cycles of neo-adjuvant chemotherapy followed by surgical resection” in the neoadjuvant arms.
- “surgical resection completed by four or more cycles of adjuvant chemotherapy” in the control arm.

Hence, this parameter represents an objective evaluation in the control arm (no neoadjuvant chemotherapy) and the two neoadjuvant chemotherapy arms (mFOLFIRINOX & FOLFOX).

### Secondary endpoints

- One and 3-year DFS (DATECAN consensus definition for the pancreas – DFS is defined by time after treatment during which no disease is found with respect to study follow-up) [42, 43]
- 3-year OS. OS is defined as the interval between the randomization date and the date of death from any cause.
- HrQoL (EORTC QLQ C-30 and QLQ-PAN26 questionnaires) The QLQ-PAN26 module is a specific module for pancreatic cancer, currently in phase IV validation by EORTC [44].
- Chemotherapy-related toxicity, graded according to CTCAE V4.0.
- Overall postsurgical morbidity, graded according to Dindo/Clavien classification [45],
- Specific morbidity due to pancreatic fistula, graded according to the International Study Group of Pancreatic Fistula (ISGPF) criteria [46].
- Histological tumor response (according to the College of American Pathologists (CAP) grading system)
- Radiological tumour response (according to RECIST v1.1) will be studied. Radiological data will be stored on a dedicated platform.
- Constitution of tissue and serum banks.

### Eligibility criteria

- Inclusion criteria

For inclusion in the study, all of the following inclusion criteria must be fulfilled:

- ○ Signed and dated informed consent
- ○ Patients willing and able to comply with protocol requirements.
- ○ Histology-proven, adenocarcinoma of the pancreas.
- ○ Resectable adenocarcinoma (according to NCCN classification 2017) [9]: the absence of distant organ or distal lymph node metastases, the absence of evidence of SMV and PV distortion, tumour thrombus, or venous encasement, the existence of clear fat planes around the celiac axis, hepatic artery and superior mesenteric artery (SMA). Resecability is evaluated on arterial-phase and portal-phase IV contrast-enhanced multislice CT-scan of the pancreas (slice thickness: 2.5 mm) and Diffusion Weighted MRI of liver, as evaluated in a multidisciplinary staff meeting including at least one radiologist and one expert surgeon.
- ○ No prior chemotherapy.
- ○ Age ≥ 18 or < 85 years
- ○ ECOG performance status 0–1.
- ○ Adequate hematologic function: neutrophils > 1.5 × 109/L; platelets > 100 × 109/L; hemoglobin ≥10 g/dL (transfusions are authorized).
- ○ Adequate renal function: creatinine clearance (according to Cockroft and Gault’s equation) > 60 ml/min
- ○ Adequate liver function: AST (SGOT) and ALT (SGPT) ≤ 2.5 x ULN (≤5 x ULN in case of liver metastases), total bilirubin ≤1.5 x ULN.
- ○ Baseline evaluations performed before randomization: clinical and blood evaluations no more than 14 days prior to randomization, tumor assessment (thorax-abdominal-pelvis CT-scan and liver MRI) no more than 21 days prior to randomization.
- ○ Women of child-bearing age not having undergone a hysterectomy or tubal ligation must undergo a negative pregnancy test (i.e. normal serum or urine beta-HCG level) before inclusion, and should use effective contraception throughout the study and for the following 6 months.

- Exclusion criteria

Patients are not eligible for this study if any of the following criteria apply:

- ○ PDAC defined as “borderline”, locally advanced non-resectable or metastatic.
- ○ Prior cancer therapy for PDAC
- ○ Surgical or anaesthesiological contra-indications:
  - ○ non-controlled congestive heart failure - non-treated angina – recent myocardial infarction (in the previous year) – non-controlled arterial hypertension (SBP > 160 mm or DBP > 100 mm, despite optimal drug treatment), long QT.
  - ○ major non-controlled infection.
- ○ Any medical, psychological or social situation that (in the investigator’s opinion) could limit (i) the patient’s compliance with the protocol or (ii) the ability to obtain or interpret data.
- ○ History or current evidence on physical examination of central nervous system disease or peripheral neuropathy ≥ grade 1, according to according to Common Terminology Criteria for Adverse Events (CTCAE) v.4.0.
- ○ Known hypersensitivity reaction to any of the components of study treatments.
- ○ Pregnancy (the absence of which must be confirmed in a ß-hCG test) or breast-feeding.
- ○ Any significant disease which, in the investigator’s opinion, would exclude the patient from the study.
- ○ Patients having been included in a clinical trial within the previous 4 weeks or participating in another trial.

### Randomization

After completion of all the screening evaluations (compliance with all the inclusion criteria and none of the exclusion criteria) and signature of the informed consent forms, all eligible patients will be randomly assigned (2:2:1) to the three arms using minimisation technique, as follows:

- The “mFOLFIRINOX” arm: neo-adjuvant chemotherapy – surgical resection– adjuvant chemotherapy
- The “FOLFOX” arm: neo-adjuvant chemotherapy – surgical resection– adjuvant chemotherapy
- The “Standard” arm: immediate surgical resection, followed by adjuvant chemotherapy (according to current guidelines)

Centralized randomization using minimisation technique will be stratified according to the study centre, the topography of tumor (uncinate/head/neck versus body/tail), bilirubin level (< 1.5 N vs. > 1.5 N) and the CA19–9 level (≤200 U/ml vs. > 200 U/ml).

### Enrolment

One hundred sixty evaluable patients need to be enrolled and randomized in a 2:2:1 ratio, with 64 patients in both the mFOLFIRINOX and FOLFOX arms, and 32 patients into the control arm.

Assuming a 5% dropout rate a total of 168 patients need to be recruited to attain the necessary power for the statistical analyses.

The accrual duration will be of 24 months.

### Ethics

This study is conducted in accordance to the standards of Good Clinical Practice (ICH-E6), the European Directive 2001/20/EC, the revised version of the Declaration of Helsinki, and local regulations. The protocol has been submitted and approved by the Agence Nationale de Sécurité du Médicament et des produits de santé (ANSM; French National Agency for Medicines and Health Product Safety) and the Comité de Protection des Personnes – NORD OUEST I (French Ethics Committee). Written informed consent is obtained from all patients prior to randomization.

### Pre-therapeutic workup

Prior to inclusion in the trial an information booklet will be given to each patient and the trial protocol will be clearly explained. The initial work-up must take place in the 4 weeks preceding randomization, and the maximum time between starting procedures and randomisation will be 21 days.

The screening assessments (this initial work-up) will comprise:

- A complete physical examination
- Biological assessments. Estimation of full blood count, calculation of creatinine clearance using the Cockroft formula, measurements of electrolytes, total protein, albumin, alkaline phosphatase, aspartate transaminase, alkaline transaminase, total and conjugated bilirubin, gamma-GT, pregnancy test for females of reproductive years and discussion of contraceptive methods during the study period
- Tumor markers evaluation: Carbohydrate antigen 19–9
- Assessment of operability – Electrocardiogram, cardiac echography, anaesthetic consultation.
- CT scan: Patients eligible for this study have resectable PDAC as shown on CT scan of thorax, abdomen and pelvis with IV contrast. As recommended by the 2015 NCCN Clinical Practice Guidelines in Oncology, the CT scan should be performed according to a defined pancreas protocol (such as three-phase cross-sectional imaging with thin slices). This technique allows accurate visualisation of the relationship between the primary tumour and the mesenteric vasculature. To this end, we shall use an optimal multiphase imaging technique that includes a non-contrast phase plus arterial, pancreatic parenchymal and portal venous phases of contrast enhancement with thin slices (2.5 mm) through the abdomen. The Pre-operative CT-scan should have been done during the 30 days period before the exact date of surgery. We shall use the latest NCCN radiological criteria (NCCN 2017) [9].
- Diagnostic confirmation by echoendoscopy with fine needle aspiration. Histopathologic confirmation of PDAC diagnosis is required before randomization. Systematic endoscopic ultrasound with fine needle aspiration or fine needel biopsy will be performed. The technique will be repeated in the event of initial negative results.
- Liver Diffusion Weighted MRI: MRI is mandatory for the detection of hepatic lesions undiagnosed by CT. By adding hepatic MRI during preoperative evaluation, hepatic metastases were newly discovered in 5% patients (5%) without hepatic lesions on CT scan [47, 48].
- Staging laparoscopy is recommended, especially if certain unfavorable prognostic factors are highlighted (*T* ≥ 3 cm CA 19–9 ≥ 200 U/ml tumor body or tail of the pancreas, back pain; greater than 10% weight loss).

During this visit, inclusion and exclusion criteria will be checked and validated. Each part of the screening evaluation will be reported on a specific form (endoscopic, clinical, biological and radiological data). Informed consent forms for the clinical study and translational research (blood samples) will be handed out to the patient. The following assessments and procedures will be performed and documented:

### Treatment methods and application

After completion of all the screening evaluations (compliance with all the inclusion criteria and none of the exclusion criteria) and signature of the informed consent forms, all eligible patients will be randomly assigned (2:2:1) to the three arms, as follows:

***ARM 1*** ***Neoadjuvant mFOLFIRINOX***: neo-adjuvant chemotherapy – surgical resection– adjuvant chemotherapy

- ***Step 1*** ***: Neoadjuvant mFOLFIRINOX***

Must start within 21 days of randomization, if bilirubin level is below 1.5 N.

Biliary drainage will be performed before the first cycle of chemotherapy administration, if bilirubin level is above 1.5 N in the mFOLFIRINOX arm. If drainage fails despite an additional endoscopic procedure, the neoadjuvant sequence will be abandoned.

4 cycles of mFOLFIRINOX neo-adjuvant chemotherapy.

Clinical and biochemical assessments (CA 19–9 assay and collection of a serum samples will be performed after the two first cycles and within 28 days of the end of the 4th cycle.

Radiological assessment (CT scan of the thorax, abdomen and pelvis) within 28 days of the end of the 4th cycle.

- ***Step 2*** ***: Surgery***

Performed 3 to 5 weeks after the end of the last cycle of neoadjuvant chemotherapy.

Pancreatic tumour resection according to current French guidelines

- ***Step 3***: ***Adjuvant chemotherapy***

Adjuvant chemotherapy for 4 months; the choice of drug or combination of drugs will be left to the medical teams, according to current guidelines and practice validated during the recruitment period.

***ARM 2*** ***Neoadjuvant FOLFOX***: neo-adjuvant chemotherapy – surgical resection– adjuvant chemotherapy

- ***Step 1*** ***: Neoadjuvant FOLFOX***

Must start within 21 days of randomization, if bilirubin level is below 1.5 N.

Even though biliary drainage is not mandatory in the FOLFOX arm, it is advisable and the decision will be left to the attending physicians.

4 cycles of FOLFIRINOX neo-adjuvant chemotherapy.

Clinical and biochemical assessments (CA 19–9 assay and collection of a serum samples will be performed after the two first cycles and within 28 days of the end of the 4th cycle.

Radiological assessment (CT scan of the thorax, abdomen and pelvis) within 28 days of the end of the 4th cycle.

- ***Step 2*** ***: Surgery***

Performed 3 to 5 weeks after the end of the last cycle of neoadjuvant chemotherapy.

Pancreatic tumour resection according to current French guidelines

- ***Step 3***: ***Adjuvant chemotherapy***

Adjuvant chemotherapy for 4 months; the choice of drug or combination of drugs will be left to the medical teams, according to current guidelines and practice validated during the recruitment period.

***ARM 3*** ***UPFRONT SURGERY***: Immediate surgical resection, followed by adjuvant chemotherapy (according to current French guidelines)

- ***Step 1*** ***: Surgery***

Performed within 21 days of randomization.

Pancreatic tumour resection according to current French guidelines

- ***Step 2*** ***: Adjuvant chemotherapy***

Adjuvant chemotherapy for 6 months; the choice of drug or combination of drugs will be left to the medical teams, according to current guidelines and practice validated during the recruitment period.

**Fig. 1:**
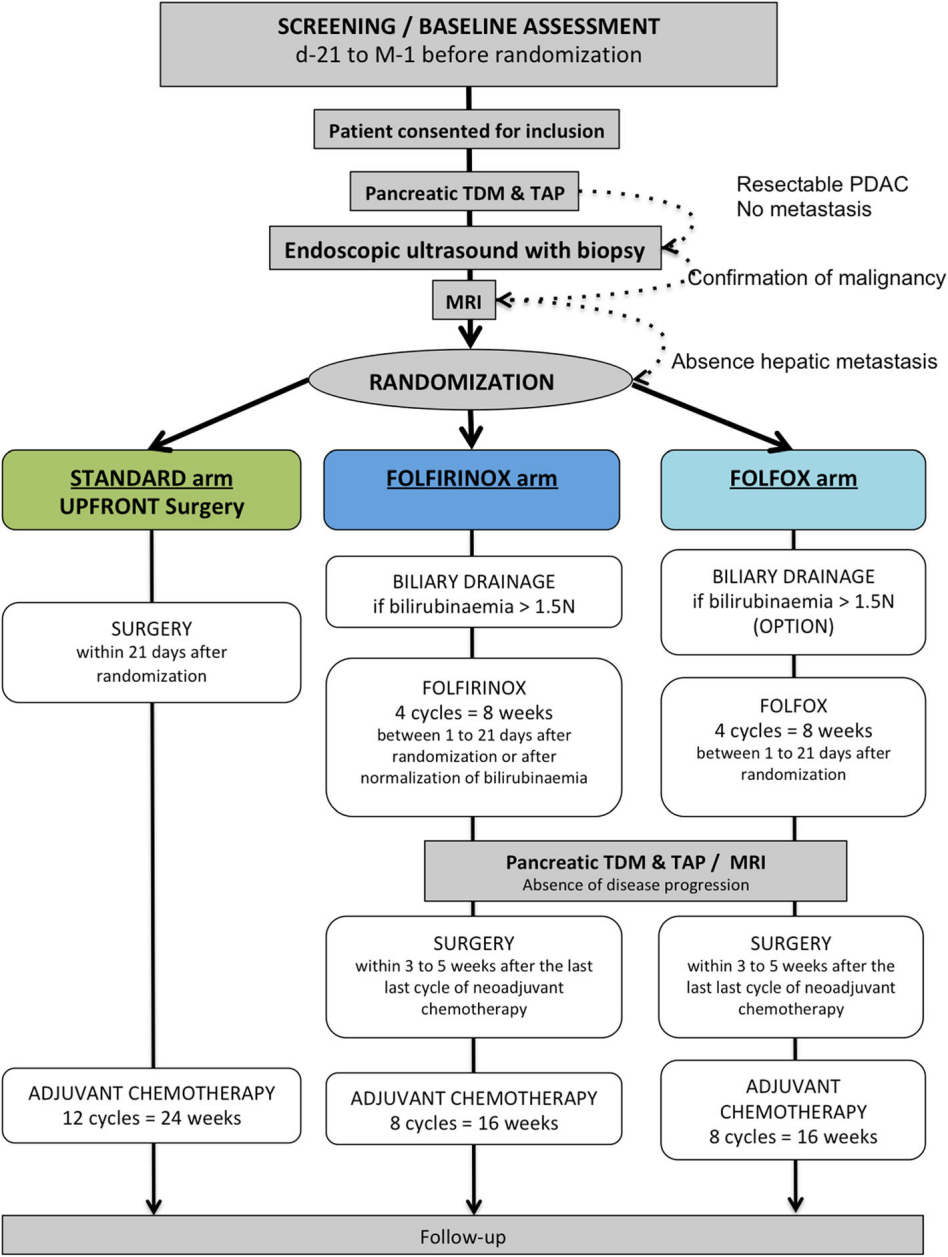
Protocol overview. MRI: Magnetic resonance imaging; PDAC: Pancreatic duct adenocarcinoma; TDM TAP: CT-scan Thorax Abdomen and Pelvis

The therapeutic scheme for this trial is depicted in Fig. 1.

### Key point of biliary drainage

Patients with bilirubin level greater than 250 micromol/l or cholangitis at presentation required systematic biliary drainage, based on the consensual indications of pre-operative biliary drainage before any surgical procedure or chemotherapy [49]. It is well accepted that the optimal duration of biliary drainage is 4 to 6 weeks. Endoscopic pre-operative biliary drainage during endoscopic retrograde cholangiopancreatography with coated self-expanded metal stents is recommended [50]. Percutaneous transhepatic biliary drainage is an option after failure of endoscopic procedure.

Otherwise, eligible patients will be randomized to either surgery alone or neoadjuvant chemotherapy.

If bilirubin level is greater than 1.5 N in patients randomized to the FOLFIRINOX arm, drainage will be implemented in all cases.

Even though biliary drainage is not mandatory in the FOLFOX arm, it is advisable and the decision will be left to the attending physicians.

### Chemotherapy regimens

Side-effects of chemotherapy are graded by the “Common Terminology Criteria for Adverse Events” version 4 https://evs.nci.nih.gov/ftp1/CTCAE/About.html

At each study visit, laboratory parameters are determined for dose adjustments.

- Neoadjuvant (pre-operative chemotherapy)
  - ○ mFOLFIRINOX Group

The modified FOLFIRINOX regimen (mFOLFIRINOX) will be administered intravenously over 48 h once every 2 weeks, for 2 months (i.e. on D1/D2, D15/D16, D29/D30 and D43/D44).

The mFOLFIRINOX regimen will be administered as follows: oxaliplatin will be administered as an 85 mg/m^2^ intravenous infusion over 2 h (on D1 only) concomitantly with irinotecan (as an 180 mg/m^2^ intravenous infusion over 90 min (on D1 only)) and LV (as a 400 mg/m^2^ infusion over 2 h), followed by 5-FU (given as a 2400 mg/m^2^ continuous infusion over 48 h, without previous bolus injection).

The cycle length is 2 weeks, comprising 48 h of infusion and 12 days of rest. These cycles must be repeated every second week. A total of 4 preoperative cycles will be administrated to all patients in the mFOLFIRINOX arm.

- ○ FOLFOX Group

The FOLFOX regimen will be administered intravenously over 48 h once every 2 weeks, for 2 months (i.e. on D1/D2, D15/D16, D29/D30 and D43/D44).

The FOLFOX regimen will be administered as follows: oxaliplatin will be administered as an 85 mg/m^2^ intravenous infusion over 2 h (on D1 only) concomitantly with LV (as a 400 mg/m^2^ infusion over 2 h), followed by 5-FU (given as a 400 mg/m^2^ bolus injection over 10 min, and then as a 2400 mg/m^2^ continuous infusion over 46 h).

The cycle length is 2 weeks, which comprises 48 h of infusion and 12 rest days. These cycles must be repeated every second week. A total of 4 preoperative cycles will be administered to all patients in the FOLFOX arm.

- ○ Re-staging post neoadjuvant chemotherapy

Tumour restaging will be performed within 28 days of the end of the 4th cycle of neoadjuvant chemotherapy. It will comprise: clinical examination, same blood analyses as at inclusion, tumour marker estimation (CA19.9), anaesthetic consultation, CT-scan of the thorax, abdomen and pelvis, and liver Diffusion Weighted MRI. This re-evaluation will be performed by the same team and by the same technique as the initial work-up.

### Surgical resection

The choice of the surgical approach will be left to the medical teams according to their experience in laparoscopic pancreatic surgery. Surgery must be performed 3 to 5 weeks after the end of the last cycle of neoadjuvant chemotherapy or as soon as possible in the control arm.

Surgical resection will follow the French national guidelines for GA, published by the SFCD-ACHBT-HAS-INCA [51].

- Pancreaticoduodenectomy

During pancreaticoduodenectomy, a para-aortic lymph node sampling is required, and should be evaluated using frozen-section analysis. Circumferential dissection of the portal vein–superior mesenteric vein (PV-SMV) axis and dissection of the right hemicircumference of the superior mesenteric artery (SMA) to the right of the coeliac trunk were required. The surgeon clearly identified the margins in the operative room with multicolour coded inking of: (i) the mesenterico–portal vein groove or PV-SMV margin (PV-SMVm); (ii) the SMA margin (SMAm), and (iii) the posterior margin.

“En-bloc” standard lymphadenectomy will be performed systematically including peripancreatic nodes (groups 13 and 17), nodes along the hepatic artery (group 8), hepatoduodenal ligament nodes (group 12), supra- and infrapyloric nodes (groups 5 and 6) and periadventitial dissection of the right lateral aspect of the SMA (groups 14b and 14c).

- Distal pancreatectomy

In the treatment of left-sided pancreatic cancer, radical antegrade modular pancreatosplenectomy (RAMPS) should be preferred [52] .

Resection of lymph nodes along the splenic artery (group 11), at the splenic hilum (group 10), and along the inferior margin of the pancreas (group 18) is recommended during distal pancreatectomy.

### Pathological analysis

A standard nomenclature should be used to describe surgical margins. For pancreatoduodenectomy specimens, the status of the pancreatic neck margin, bile duct margin, anterior surface, posterior surface, portal vein/SMV groove, and SMA/uncinate margin should be described. For distal pancreatectomy specimens, the anterior surface, posterior surface, and pancreatic transection margins should be described. The pathological protocol also included the maximal transverse diameter of the tumour, the distance between the tumor and the different surgical margins (mm) the tumour–node–metastasis (TNM) classification, the grade of differentiation, the presence or absence of perineural, lymphatic and/or vascular spread, the number of lymph nodes retrieved from the specimen, enabling the calculation of the lymph node ratio (LNR) and the pathological tumor response rate (according to the CAP grading system).

Serial slicing of the entire pancreatic specimen will be performed according to the guidelines of the Royal College of Pathologists and the Leeds Pathology Protocol [12, 53]. Margin involvement (R1) is defined for the 0-mm margin if tumour cells were present at the inked margin; R1 is also defined for each margin width if tumour cells were present within the margin, independently of the mode of tumour spread; The resection was considered as curative (R0) if no tumour cells were identified at any of the resection margins, again for each margin width.

### Patient follow-up

After surgery, participating patients will be reviewed in out-patient consultations 1 month (+/− 14 days) after hospital discharge. They will have a full clinical examination, biochemical analysis, tumour marker estimation (CA19.9), thorax-abdominal-pelvis CT scan. Questionnaires will be completed with regards to quality of life (QoL), emotional state, emotional adjustment, perception of illness and lifestyle burden.

After completion of adjuvant chemotherapy, a further consultation will be scheduled for one month (+/− 14 days) with evaluation of clinical state, treatment tolerance, haematological parameters, renal function, tumour markers, and the same set of questionnaires will be completed.

All participants will subsequently be followed for 3 years after randomization. Out-patient consultations will be scheduled every 3 months (+/− 15 days) and patients will be evaluated by clinical examination, thoraco-abdominal-pelvic CT scan, tumour marker estimation (CA19.9) and QoL questionnaires.

Further examinations will be requested as required.

If disease recurrence is suspected its site (loco regional or distant) must be documented by the appropriate investigations and histological proof be obtained wherever possible. In cases of strong clinical suspicion of recurrence without radiological or histological proof, surgical exploration is recommended to identify peritoneal carcinomatosis not visualised by standard radiological investigation. All cases of disease recurrence will be discussed at the multi-disciplinary team meeting and further therapeutic strategies recorded.

### Reason for discontinuing treatment protocol

The treatment protocol may be discontinued for the following reasons: decision of the treating doctor, high grade toxicity, occurrence of a serious adverse effect, disease progression, patient refusal to continue and patient death. In cases where treatment is discontinued, patients will remain within the intention to treat analysis.

### Samples size calculation and statistical considérations

According to Bryant and Day two-stage design [54] with a ration of 2:2:1, it will be required to randomize 64 patients into the FOLFOX arm, and 64 patients into the FOLFIRINOX arm (with a one-sided type 1 error of 5% and a power of 85%) to verify the following hypotheses:

- Hypothesis H0: an observed OS rate at 12 months of 70% and a feasibility of the full therapeutic sequence in 55% of patients in the neo-adjuvant FOLFOX and FOLFIRINOX groups will be considered as uninteresting to pursue evaluation in Phase III
- Hypothesis H1: The expected outcome corresponds to an observed OS rate at 12 months of 85% and a feasibility in 75% of patients in the neo-adjuvant FOLFOX and FOLFIRINOX groups

In first stage, an intermediate analysis will be performed once 27 evaluable patients with 12 months of follow-up have been included in each of the 2 arms.

- Upper limit for rejecting drug interest due to inadequate OS rate at 12 months will be 19 deaths
- Upper limit for rejecting drug interest due to inadequate feasibility toxicity will be 15 patients without full therapeutic sequence

In other case we will pursue the inclusion by including 37 additional patients in each arm reaching the criteria for a total of 64 evaluable patients

- Upper limit for rejecting drug interest due to inadequate OS rate at 12 months will be 50 deaths
- Upper limit for rejecting drug interest due to inadequate feasibility toxicity will be 41 patients without full therapeutic sequence

In other case treatment will considered as promising and will be regarded as interesting for further evaluation in a phase III trial.

Taking into account 32 additional patients to be randomized in the control arm to validate our hypotheses (especially H0) due to the 2:2:1 randomised ratio, 160 patients should be randomized.

Taking in account, 5% of patients lost to follow up 168 patients will need to be enrolled.

In case of positive results of the phase 2 we plan to continue with a phase III trial at the end of the phase II. The phase 2 will then constitute an interim analysis of phase III (O Brien Fleming boundaries with alpha spending function) in this case statistical comparison will be done in the line of phase III design with stringent marging to reject earlier H0 or H1 (futility), such results would not be communicated in case of no rejection. Patients randomized and included in the phase II will be kept for phase III according to phase II results.

A statistical analysis plan will be written before data frozen. All changes to this plan will be documented. Since this a non comparative randomized phase II trial, statistical comparisons will be done in a exploratory purpose.

## Discussion

The present trial offers the unique opportunity to explore the interest of 2 neoadjuvant chemotherapy regimens (FOLFOX, mFOLFIRINOX) in contrast of immediate surgery with adjuvant chemotherapy in the setting of resectable PDAC.

### Neoadjuvant strategy in resectable PDAC

In theory, neoadjuvant treatment has several potential advantages in the context of resectable PDAC.

In terms of the current standard management of surgery with adjuvant therapy, between 40 and 50% of patients having undergone curative resection do not receive the adjuvant treatment planned due to surgical complications, poor performance status, comorbidity, patient refusal, and/or early disease recurrence [21–23, 55–58]. Otherwise, completion of the full therapeutic sequence after neoadjuvant treatment was observed in 75% in a recent report from an experienced centre [21]. Hence, neoadjuvant strategy may allow administrating the full therapeutic sequence to a greater proportion of patients than adjuvant strategy alone.

Neoadjuvant therapy may achieve down-staging, which in turn improves the R0 resection rate and increases the locoregional control. Indeed, Schorn reported that after neoadjuvant therapy, the rates of vascular and perineural involvement were lower than for immediately resected tumours [59]. Moreover, significantly lower positive lymph node (LN) rates were observed after neoadjuvant treatment (between 10 and 40%) [27, 30, 59] whereas lymph node involvement is constant in case of upfront surgery - between 65 and 80% of cases [60, 61].

Although complete resection of these “resectable” tumours would seem to be technically possible, the actual R1 resection rate is liable to be high. As mentioned above evoked, a recent prospective analysis of pancreatic resection specimens (using a standardized pathological reporting protocol) has demonstrated that according to the “redefinition” of margin status, the frequency of positive-margin resection (R1) is as high as 70%. The R1 resection rate was 60% in the ESPAC-4 trial [14]. This value provides a rationale for neoadjuvant treatment, with the hope of improving the rate of R0 resection.

Indeed, recent studies on neoadjuvant therapy in a context of borderline resectable disease, confirmed this hypothesis, with regard to achievement of R0 resection in more than 90% of cases [34, 36, 62].

The concern for disease progression during neoadjuvant treatment is not negligible, due to the low rate of curative resections performed in patients whose disease was deemed resectable at the time of starting neoadjuvant therapy - both after induction chemoradiation (45–74%) [25, 30, 31] and after induction chemotherapy (38–70%) [28, 63]. However, advocates of neoadjuvant therapy claim that these data show the value of this strategy and argue that patients who experience disease progression during induction treatment are suffering from an extremely aggressive tumour and undiagnosed initial micrometastatic disease that cannot be cured by extensive surgery. This type of strategy may help select good candidates for surgery. It would be desirable to reduce or avoid the risk of surgical mortality and morbidity in this subset of patients.

### Which neoadjuvant chemotherapy?

Even though single-agent gemcitabine and 5FU have been validated in adjuvant and metastatic settings, the objective response was low, at around 10% [64, 65], whereas combination chemotherapy yielded a response rate of 9–28% in advanced PDAC [66–70]. Interestingly, using more than two chemotherapeutic agents in advanced PDAC increased the response rate: the three-drug combinations were gemcitabine, a fluoropyrimidin and a platinating agent (18–41%) [71–74], either FOLFIRINOX (5-FU-oxaliplatin-irinotecan; 27–50%) [75–78] or G-FLIP (gemcitabine-5FU-irinotecan-cisplatin; 26%) [79] and showed promising results.

The superiority of intensive polychemotherapy regimens was also suggested by an Italian survey of treatment trends and outcomes in large series of patients with stage III/IV PDAC [80]. Gemcitabine alone appeared to be significantly inferior to four-drug combinations, with median OS times of about 5.1 and 9.1 months, respectively. Based on these data and considerations, polychemotherapy regimens are suitable candidates in a neoadjuvant setting.

Moreover, even though gemcitabine is still one of the main weapons in the oncologist’s arsenal, recent studies have shown that two thirds of patients with PDAC do not benefit from gemcitabine based-chemotherapy in an adjuvant setting because of low or moderate expression levels of gemcitabine nucleoside transporters (hENT1) [81–83]. In this context, it will be interesting to more accurately define the subgroups of patients who would derive particular benefit from gemcitabine and those who should be treated with non-gemcitabine-based combinations. A pre-operative assay of hENT1 expression in biopsy samples is not currently feasible in multicentre trial even if studies are ongoing [84]. Hence, from an ethical point of view, we decided to compare two non-gemcitabine-based combinations: mFOLFIRINOX and FOLFOX.

### mFOLFIRINOX

In 2005, Conroy et al. [75] evaluated the response rate and toxicity of FOLFIRINOX in an advanced PDAC setting. The study yielded promising results, with a response rate of 27%, a time to progression of 8.2 months, and an OS of 10.2 months. Despite the fact that grade 3/4 neutropenia occurred in 52% of patients, patients had improvement in all functional scales of the EORTC QLQ-C30 assessment questionnaire. Based on these data, Conroy and colleagues conducted a phase III trial comparing FOLFIRINOX with gemcitabine as first-line treatment for metastatic PDAC in 342 patients with good performance status (0–1) [76]. Median OS was 11.1 months in the FOLFIRINOX group and 6.8 months in the gemcitabine group (hazard ratio (HR): 0.57; 95% confidence interval (CI): 0.45–0.73; *p* < 0.001). Median progression-free survival (PFS) was 6.4 months vs. 3.3 months (HR: 0.47; 95% CI: 0.37–0.59, *p* < 0.001). The overall response rate was 31.6% vs. 9.4% (*p* < 0.001). Grade 3/4 toxicities were more common in the FOLFIRINOX group than in the gemcitabine group (diarrhoea 12.7% vs. 1.8%, nausea 15.6% vs. 6.3%, vomiting 14.5% vs. 8.3%, fatigue 23.6% vs. 17.8%, neutropenia 45.7% vs. 21%, and febrile neutropenia 5.4% vs. 1.2%, respectively). In the FOLFIRINOX arm, 42% of patients received support with granulocyte colony-stimulating factor.

One of the main criticisms of this trial was that only 39% of patients had a primary tumour in the head of the pancreas (possibly requiring biliary stents), whereas the proportion is about two-thirds in clinical practice. This type of polychemotherapy can cause severe neutropenia, which is a major concern in this setting because of the specific risk of cholangitis in patients with biliary stents. However, recent data suggest that after effective biliary drainage, FOLFIRINOX could be used even for locally advanced PDAC patients with jaundice, and is currently proposed as first line therapy for Borderline and Locally advanced PDAC [9, 85]. In the studies by Marthey et al. [78] and Hosein et al. [86], respectively 47 and 50% of patients required biliary drainage. No patient was taken off FOLFIRINOX for toxicity reasons, even though 26 and 40% respectively experienced grade 3/4 adverse events. This toxicity profile was managed by use of a granulocyte colony-stimulating factor analogue in up to 85% of cases and a FOLFIRINOX dose reduction in 20% of cases in both studies [78, 86]. The toxicity profiles did not appear to depend on the presence or absence of a stent. There was no episode of cholangitis during FOLFIRINOX treatment in any of the patients. Other recently published retrospective studies have shown similar results [77, 87], confirming that FOLFIRINOX could be a viable option even in patients with a biliary stent. Therefore, criteria such as good performance status, controlled bilirubin level and good supportive care must be complied with.

Given the toxicity of FOLFIRINOX, there is a pressing need for active, safe agents - especially for patients with poor performance status and who cannot risk exposure to the high toxicity of a combined regimen.

Modified FOLFIRINOX was better tolerated and as efficient as “classic” FOLFIRINOX in previous studies [84]. Moreover, the tolerability and feasibility of mFOLFIRINOX has been demonstrated in a phase I-II trial in patients with borderline PDAC [63].

### FOLFOX

The oxaliplatin–5-FU-based combination also appears to be a valuable option. One of the oxaliplatin/5FU combinations, the FOLFOX 6 regimen, has shown a good response rate in pancreatic cancer [88, 89], with tolerable rates of grade 3/4 toxicity. In 2012, n et al. confirmed their promising results for the oxaliplatin/5FU combination regimen in a prospective phase II trial that sought to evaluate sequential FOLFOX-6 and gemcitabine followed by appropriate maintenance treatment for advanced pancreatic cancer. The clinical response rate and biochemical response rate (based on significant decrease in CA 19–9 levels) were 44% (22% for both the partial response and stable disease) and 64.3%, respectively. The toxicity profile of the FOLFOX-6 combination was acceptable and manageable [40].

Recently, the NCCN stated that Fluoropyrimidines plus oxaliplatin combination therapy can be an acceptable option for patients with gemcitabine refractory pancreatic cancer [9]. Several studies have reported infused 5-FU plus oxaliplatin as a second line treatment with a combined response and disease control rate of 17 to 52%, PFS of 1.4 to 4.0 months and OS of 3.4 to 6.7 months [90–94].

The main objective of our Phase II PANACHE-01 study is to assess the safety and efficacy of neo-adjuvant mFOLFIRINOX and FOLFOX in patients with resectable PDAC, relative to the current reference treatment (surgery and then adjuvant chemotherapy). The control arm will consist of patients treated with upfront surgery followed by adjuvant chemotherapy. This study will enable us to investigate the efficacy and tolerability of a neo-adjuvant treatment protocol and to select the best regimen for improving rates of long-term DFS and complete/sustained remission in these patients.

The most innovative aspect of this study would be its validation of the neoadjuvant approach as treatment for resectable PDAC, with the use of aggressive drugs (FOLF(IRIN)OX). To date neoadjuvant FOLF(IRIN)OX treatment has not been tested for resectable tumours.

In view of the limitations of the reference treatment (surgical resection followed by adjuvant chemotherapy) in terms of post-operative feasibility (fewer than 60% of patients can be treated) and lack of efficacy (in term of resection margins, node involvement, DFS and OS), other approaches must be considered.

The “ideal” cancer treatment for resectable PDAC would have the following characteristics: administration to the highest possible proportion of patients, ability to identify fast-progressing patients (i.e. poor candidates for surgery), a low proportion of R1 resections (through optimisation of local disease control), and an acceptable rate of toxicity profile. The neo-adjuvant approach meets these criteria and has been validated (or is being evaluated) for other main digestive tract sites (the rectum, stomach, oesophagus and colon). With respect to published data on the efficacy of FOLFOX and mFOLFIRINOX, these two combination regimens are potential candidates for neo-adjuvant use with a view to optimize oncological outcomes in resectable PDAC.

## Abbreviations

DFS: Disease free survival
HRQoL: Health-related quality of life
OS: Overall survival
PDAC: Pancreatic duct adenocarcinoma
PV: Portal vein
R0: Disease free margin
SMA: Superior mesenteric artery
SMV: Superior mesenteric vein
