# Correction to: Resectable pancreatic adenocarcinoma neo-adjuvant FOLF(IRIN)OX-based chemotherapy - a multicenter, non-comparative, randomized, phase II trial (PANACHE01-PRODIGE48 study)

**DOI:** 10.1186/s12885-020-6678-x

**Published:** 2020-03-03

**Authors:** Lilian Schwarz, Dewi Vernerey, Jean-Baptiste Bachet, Jean-Jacques Tuech, Fabienne Portales, Pierre Michel, Antonio Sa Cunha

**Affiliations:** 1 Department of Digestive Surgery, Hôpital Charles Nicolle, Rouen, France; 2 UNIROUEN, UMR 1245 INSERM, Rouen University Hospital, Department of Genomic and Personalized Medicine in Cancer and Neurological Disorders, Normandie Univ, F-76000 Rouen, France; 3 Methodological and Quality of Life in Oncology Unit, INSERM UMR 1098, University Hospital of Besançon, Besançon, France; 4 Department of Hepato-Gastroenterology, Hôpital Pitié Salpêtrière, Paris, France; 5 Department of Digestive Oncology, Institut régional du Cancer de Montpellier (ICM), Val d’Aurelle, Montpellier, France; 6 Department of Hepato-Gastroenterology, Rouen University Hospital, Rouen, France; 7 Department of Hepatobiliary and Pancreatic Surgery and Liver Transplantation, Paul Brousse Hospital, Villejuif, France

**Correction to: BMC Cancer (2018) 18:762**

**https://doi.org/10.1186/s12885-018-4663-4**

Following publication of the original article [1], the authors reported an error in the “Samples size calculation and statistical considerations” section.

The corrected section is presented below:

**Samples size calculation and statistical considerations**

According to Bryant and Day two-stage design [54] with a ratio of 2:2:1, it will be required to randomize 64 patients into the FOLFOX arm, and 64 patients into the FOLFIRINOX arm (with a one-sided type 1 error of 5% and a power of 85%) to verify the following hypotheses:

- Hypothesis H0: an observed OS rate at 12 months of 70% and a feasibility of the full therapeutic sequence in 55% of patients in the neo-adjuvant FOLFOX and FOLFIRINOX groups will be considered as uninteresting to pursue evaluation in Phase III
- Hypothesis H1: The expected outcome corresponds to an observed OS rate at 12 months of 85% and a feasibility in 75% of patients in the neo-adjuvant FOLFOX and FOLFIRINOX groups
- In first stage, an intermediate analysis will be performed once 27 evaluable patients for OS at 12 months and for the feasibility of the therapeutic sequence have been included in each of the 2 arms.
- If 19 (70.4%) or less than 19 patients are alive at 12 months no more additional patient will be included in the arm. i.e. 8 death or more
- If 15 (55.5%) or less than 15 patients are identified with full therapeutic sequence no more additional patient will be included in the arm. i.e. 12 patients without full therapeutic sequence or more

In other case we will pursue the inclusion by including 37 additional evaluable patients in each arm reaching the criteria for a total of 64 evaluable patients

- If 50 (78.1%) or less than 50 patients are alive at 12 months the treatment will be declared uninteresting. i.e. 14 death or more
- If 42 (65.6%) or less than 42 patients are identified with full therapeutic sequence the treatment will be declared uninteresting. i.e. 22 patients without full therapeutic sequence or more

In other case treatment will considered as promising and will be regarded as interesting for further evaluation in a phase III trial.

Taking into account 32 additional patients to be randomized in the control arm to validate our hypotheses (especially H0) due to the 2:2:1 randomized ratio, 160 patients should be randomized.

Taking in account, 5% of patients lost to follow up 168 patients will need to be enrolled.

In case of positive results of the phase 2 we plan to continue with a phase III trial at the end of the phase II.

The phase 2 will then constitute an interim analysis of phase III (O Brien Fleming boundaries with alpha spending function) in this case statistical comparison will be done in the line of phase III design with stringent marging to reject earlier H0 or H1 (futility), such results would not be communicated in case of no rejection. Patients randomized and included in the phase II will be kept for phase III according to phase II results.
